# Complex Emotions in Pediatric Care: Unravelling the Challenges and Pathways for Staff

**DOI:** 10.1192/bjo.2025.10496

**Published:** 2025-06-20

**Authors:** Praveen Kumar, Tharini Kumar, Jane Hosie

**Affiliations:** 1 City Hospital, Aberdeen, United Kingdom; 2 New Craig’s Psychiatric Hospital, Inverness, United Kingdom; 3 Louth Hospital, Dublin, Ireland

## Abstract

**Aims:** The aim of this study was to explore the experiences and challenges faced by staff in pediatric wards when managing young patients with complex emotional and behavioural difficulties. It sought to understand the specific nature of these challenges, their impact on healthcare providers, and to identify potential strategies for improvement, particularly focusing on the staff’s interaction with Child and Adolescent Mental Health Services (CAMHS).

**Methods:** The study involved a qualitative survey of 16 healthcare professionals, including 12 nurses, 3 administrative or support staff, and 1 doctor, working in a Paediatric ward setting. Open-ended questions were used to gather detailed insights into the staff’s experiences. The responses were then segmented and analysed, focusing on the nature of the challenges faced, the impact on staff, suggestions for improvements, and the dynamics of their relationship with CAMHS.

**Results:** The survey revealed a multifaceted set of challenges. Staff reported a significant knowledge gap in managing patients with complex emotional issues, often leading to feelings of inadequacy and stress. These challenges were not just clinical but also emotional, affecting staff morale and mental health. The responses underscored the need for better support, specialized training, and enhanced resources. A recurring theme was the pivotal role of CAMHS, with staff expressing a need for more effective collaboration and communication. The data also hinted at nuanced challenges, such as dealing with manipulative behaviours, difficulty in patient–family interactions, and the emotional toll of such encounters. These findings highlight the complexity of emotional and behavioural management in pediatric care, extending beyond patient interaction to encompass broader aspects of the healthcare environment.

**Conclusion:** The study confirmed that Paediatric ward staff face considerable challenges in managing young patients with complex emotional difficulties. These challenges go beyond clinical management, significantly impacting the staff’s emotional well-being. The findings point to an urgent need for targeted training and support initiatives, along with stronger collaborative ties with CAMHS. Implementing such measures could lead to improved patient care and staff satisfaction. Additionally, regular debriefing sessions and feedback mechanisms are recommended to continually adapt and optimize care strategies in Paediatric wards, ensuring a resilient and empathetic healthcare environment.

